# Genetic Etiology for Alcohol-Induced Cardiac Toxicity

**DOI:** 10.1016/j.jacc.2018.03.462

**Published:** 2018-05-22

**Authors:** James S. Ware, Almudena Amor-Salamanca, Upasana Tayal, Risha Govind, Isabel Serrano, Joel Salazar-Mendiguchía, Jose Manuel García-Pinilla, Domingo A. Pascual-Figal, Julio Nuñez, Gonzalo Guzzo-Merello, Emiliano Gonzalez-Vioque, Alfredo Bardaji, Nicolas Manito, Miguel A. López-Garrido, Laura Padron-Barthe, Elizabeth Edwards, Nicola Whiffin, Roddy Walsh, Rachel J. Buchan, William Midwinter, Alicja Wilk, Sanjay Prasad, Antonis Pantazis, John Baski, Declan P. O’Regan, Luis Alonso-Pulpon, Stuart A. Cook, Enrique Lara-Pezzi, Paul J. Barton, Pablo Garcia-Pavia

**Affiliations:** a National Heart and Lung Institute, Imperial College London, London, United Kingdom; b Cardiovascular Research Centre, Royal Brompton and Harefield NHS Foundation Trust London, London, United Kingdom; c MRC London Institute of Medical Sciences, Imperial College London, London, United Kingdom; d Heart Failure and Inherited Cardiac Diseases Unit, Department of Cardiology, Hospital Universitario Puerta de Hierro, Madrid, Spain; e Institute of Psychiatry, Psychology and Neuroscience, Social Genetic and Developmental Psychiatry Centre, King's College London, London, United Kingdom; f Department of Cardiology, Hospital Universitario de Tarragona Joan XXIII, IISPV, Rovira Virgili University, Tarragona, Spain; g Inherited Cardiac Diseases Unit, Department of Cardiology, Hospital Bellvitge, Barcelona, Spain; h Genetics Department, Universidad Autónoma de Barcelona, Barcelona, Spain; i CIBER in Cardiovascular Diseases, Madrid, Spain; j Heart Failure and Inherited Cardiac Diseases Unit, Department of Cardiology, Hospital Universitario Virgen de la Victoria, IBIMA, Málaga, Spain; k Department of Cardiology, Hospital Universitario Virgen de la Arrixaca, IMIB-Arrixaca, University of Murcia, Murcia, Spain; l Cardiology Department, Hospital Clínico Universitario, INCLIVA Universitat de Valencia, Valencia, Spain; m Department of Biochemistry, Hospital Universitario Puerta de Hierro, Madrid, Spain; n National Heart Research Institute Singapore, National Heart Centre Singapore, Singapore; o Division of Cardiovascular & Metabolic Disorders, Duke-National University of Singapore, Singapore; p Myocardial Biology Programme, Centro Nacional de Investigaciones Cardiovasculares (CNIC), Madrid, Spain; q University Francisco de Vitoria (UFV), Pozuelo de Alarcón, Madrid, Spain

**Keywords:** alcohol, dilated cardiomyopathy, genetics, titin, variant, ACM, alcoholic cardiomyopathy, DCM, dilated cardiomyopathy, ExAC, Exome Aggregation Consortium, LVEF, left ventricular ejection fraction, TTNtv, titin truncating variant

## Abstract

**Background:**

Alcoholic cardiomyopathy (ACM) is defined by a dilated and impaired left ventricle due to chronic excess alcohol consumption. It is largely unknown which factors determine cardiac toxicity on exposure to alcohol.

**Objectives:**

This study sought to evaluate the role of variation in cardiomyopathy-associated genes in the pathophysiology of ACM, and to examine the effects of alcohol intake and genotype on dilated cardiomyopathy (DCM) severity.

**Methods:**

The authors characterized 141 ACM cases, 716 DCM cases, and 445 healthy volunteers. The authors compared the prevalence of rare, protein-altering variants in 9 genes associated with inherited DCM. They evaluated the effect of genotype and alcohol consumption on phenotype in DCM.

**Results:**

Variants in well-characterized DCM-causing genes were more prevalent in patients with ACM than control subjects (13.5% vs. 2.9%; p = 1.2 ×10^−5^), but similar between patients with ACM and DCM (19.4%; p = 0.12) and with a predominant burden of titin truncating variants (TTNtv) (9.9%). Separately, we identified an interaction between *TTN* genotype and excess alcohol consumption in a cohort of DCM patients not meeting ACM criteria. On multivariate analysis, DCM patients with a TTNtv who consumed excess alcohol had an 8.7% absolute reduction in ejection fraction (95% confidence interval: −2.3% to −15.1%; p < 0.007) compared with those without TTNtv and excess alcohol consumption. The presence of TTNtv did not predict phenotype, outcome, or functional recovery on treatment in ACM patients.

**Conclusions:**

TTNtv represent a prevalent genetic predisposition for ACM, and are also associated with a worse left ventricular ejection fraction in DCM patients who consume alcohol above recommended levels. Familial evaluation and genetic testing should be considered in patients presenting with ACM.

Alcoholic cardiomyopathy (ACM) is caused by chronic and excessive alcohol intake 1, 2, 3, 4. Although moderate levels of alcohol consumption may have some beneficial cardiovascular effects 3, 5, prolonged and excessive consumption can lead to deleterious consequences including cardiac arrhythmias and a dilated cardiomyopathy (DCM) phenotype 2, 3, 4, 5. The pathophysiology of ACM is not fully understood, and the relationship between the degree of alcohol exposure and severity of end-organ damage is not simple 2, 6. In particular, not all individuals with high alcohol intake develop ACM, and this heterogeneity in response indicates differences in underlying susceptibility, likely both genetic and environmental. However, studies of heritable contributors to ACM are currently limited 7, 8. Prognosis in ACM is poor, but is considered more favorable than DCM generally, with recovery observed in up to one-third of cases, especially when alcohol intake is reduced (3). Current management of ACM individuals includes cessation of alcohol exposure, standard heart failure medications, and prevention of sudden cardiac death 2, 6, 9. As the genetic contribution to ACM is currently unknown, familial evaluation is not part of ACM management.

DCM is estimated to affect up to 1 in 250 individuals (10) and has a significant genetic contribution, with truncation variants in the gene encoding titin (TTNtv), a key sarcomeric protein, representing the predominant genetic cause, seen in 10% to 20% of cases 11, 12, 13. It is also recognized that up to 1% of the general population carry a TTNtv, presenting a significant challenge to interpretation (12). Genetic and/or environmental factors likely underlie the variable penetrance and expressivity. In line with this, recent evidence has shown that over 10% of patients with peripartum cardiomyopathy carry a TTNtv, suggesting that in some patients, the DCM phenotype results from a combination of pregnancy with a genetic predisposing background (14).

Moreover, we have recently demonstrated that TTNtv found in the general population are not phenotypically silent (15); although the population prevalence of TTNtv exceeds the prevalence of DCM, careful phenotyping reveals differences in cardiac volumes in subjects with and without TTNtv. Using a rat model, we found essentially normal resting cardiac function, but subclinical metabolic abnormalities in TTNtv carriers and impaired cardiac physiology under conditions of cardiac stress (15). Together, these data suggest that TTNtv may predispose to cardiomyopathy, with environmental factors modulating penetrance and expressivity.

Here, we sought to evaluate genetic determinants in the pathophysiology of ACM by characterizing genetic variation in known DCM-causing genes in a large ACM cohort. We sequenced 141 individuals with ACM and compared these with healthy volunteers (n = 445), individuals with DCM (n = 366), and population-based variant frequency data (Exome Aggregation Consortium [ExAC]; n = 60,706). We further evaluated the phenotypic effect of excessive alcohol intake (below the levels required for a diagnosis of ACM) in the context of TTNtv in a wider cohort of 716 DCM subjects.

## Methods

The study conformed to the ethical principles of the Declaration of Helsinki and was approved by the local institutional review board of Hospital Universitario Puerta de Hierro and a National Health Service Health Research Authority Research Ethics Committee. All patients provided written informed consent.

### ACM cases

A total of 141 unrelated patients with ACM (Table 1) were recruited for this study from 6 Spanish hospitals. ACM was defined as DCM with a history of prolonged and heavy alcohol consumption: that is, a self-reported history of alcohol intake of >80 g/day over a period of at least 5 years 2, 3, 6, with excess intake continuing up to no <3 months before initial diagnosis of ACM, in combination with DCM defined by established criteria of left ventricular dilation and reduced ejection fraction in the absence of coronary artery disease (invasive or computed tomography angiographic evidence of >50% stenosis in any major epicardial coronary artery, or previous percutaneous coronary intervention or coronary artery bypass grafting) or abnormal loading conditions (uncontrolled hypertension or significant primary valvular disease). Outcome information was collected until last available follow-up, or at death or transplantation, and follow-up time was truncated at 12 years. Although a specific program for alcohol discontinuation was not provided, complete abstinence from alcohol was recommended to all ACM patients. Endpoints were pre-specified as: 1) death or cardiac transplantation; and 2) recovery defined as an absolute increase in left ventricular ejection fraction (LVEF) ≥10% to a final value of ≥40% (16). Survival analyses measured time from diagnosis (first assessment in heart failure clinic) to first event. Although genotype, which defined groups for comparison in survival analysis, was ascertained retrospectively, both clinical care and outcome adjudication were blinded to genotype.

**Table 1:** Clinical Characteristics of Patient Cohorts

	ACM (n = 141)	DCM (n = 366)	Healthy Volunteer (n = 445)
Age at scan, yrs	53.2 ± 10.0	56.0 ± 13.6	40.8 ± 13.5
Left ventricular ejection fraction (echo), %	26.5 ± 9.3	—	—
Left ventricular ejection fraction (CMR), %	—	38.7 ± 12.8	66.1 ± 5.1
Left ventricular end-diastolic diameter (echo), mm	65.6 ± 9.1	—	—
Left ventricular end-diastolic volume (CMR), ml	—	257.7 ± 82.6	149.3 ± 32.6
Males	138 (97.9)	255 (69.7)	201 (45.2)
Ethnicity (Caucasian)	141 (100.0)	366 (100.0)	445 (100.0)

Values are mean ± SD or n (%).

ACM = alcoholic cardiomyopathy; CMR = cardiac magnetic resonance; DCM = dilated cardiomyopathy.

### DCM cases

A total of 716 consecutive patients with DCM confirmed by late gadolinium enhancement cardiac magnetic resonance were prospectively enrolled in the Royal Brompton Hospital Cardiovascular Research Centre Biobank between 2009 and 2015 as previously described (17). DCM was diagnosed based on established criteria of left ventricular dilation and reduced ejection fraction with reference to age- and sex-adjusted nomograms (18) in the absence of known coronary artery disease (defined as presence of subendocardial late gadolinium enhancement suggestive of previous myocardial infarction or >50% stenosis in any major epicardial coronary artery or previous percutaneous coronary intervention or coronary artery bypass grafting) or abnormal loading conditions as for ACM. The complete 716 DCM cohort was evaluated for phenotypic correlates of *TTN* genotype and alcohol exposure (described in the following text), and a subset of 366 unrelated cases that were matched both technically and by ethnicity with the ACM cohort were used for comparative genetic analysis.

### Healthy volunteers

A total of 445 healthy volunteers free from self-reported cardiovascular disease or a family history of disease were recruited prospectively via advertisement to the U.K. Digital Heart Project at the MRC-LMS, Imperial College London (15). All participants underwent clinical assessment, including cardiac magnetic resonance, to confirm the absence of cardiac disease.

### Next-generation sequencing and variant analysis

See the Online Methods in the Online Appendix for full details on sequencing, variant filtering, and annotation. In brief, sequencing was carried out using the Illumina TruSight Cardio Sequencing kit (San Diego, California) (19) or a custom Agilent SureSelect XT target capture (Santa Clara, California) with similar content and run on Illumina platforms or Life Technologies 5500XL (Waltham, Massachusetts). Rare (ExAC filtering allele frequency [20] <8.4 × 10^−5^) protein-altering variants were identified in genes and variant classes proven to be robustly associated with DCM (Online Table 1). In the case of titin, analysis was further restricted to truncating variants in exons constitutively expressed in the heart as described (12). Although the Illumina TruSight Cardio sequencing kit captures 61 genes purportedly associated with DCM (full gene list and variants detected are given in Online Table 7), we decided to be conservative and pre-specified a focused analysis on 9 genes with the most robust evidence of disease association (*TTN*, *DSP, MYH7*, *LMNA*, *TTNT2*, *TCAP, SNC5A, BAG3,* and *TNNC1*) and compared the prevalence of rare protein-altering variants in subjects who were matched both technically (TruSight Cardio panel and NextSeq platform [both Illumina]) and by ethnicity (self-reported Caucasian, confirmed by PCA analysis [see Online Methods in the Online Appendix]). The 9 genes assessed are those with a demonstrated excess of rare variation in DCM clinical cohorts over ExAC reference samples, for either truncating or nontruncating variants 13, 21.

### Evaluation of alcohol as a phenotypic modifier in DCM

We investigated the effect of alcohol consumption on phenotype in DCM patients using self-reported weekly consumption together with a review of hospital and primary care medical records for a history of alcohol excess prior to study recruitment. No patients had a history of prolonged heavy alcohol consumption for a diagnosis of ACM. “Excess alcohol consumption” in DCM was defined as a binary variable indicating a history of consumption >21 U/week for men and >14 U/week for women (1 U of alcohol = 10 ml or 8 g of pure alcohol, an amount the average adult metabolizes in 1 h) (22), levels representing the “sensible limits” for alcohol consumption from U.K. consensus medical advice (23) from 1987 to 2016.

Univariable linear regression was performed to identify variables predictive of LVEF at study recruitment. LVEF was measured while blind to genotype. Variables with p < 0.10 in univariable analysis were included in a multivariable model, which was then optimized by reverse stepwise selection until only significant variables were included. The pre-specified main analysis assessed the significance of an interaction term between TTNtv and “excess alcohol consumption” added to this optimized multivariable model predicting LVEF, to determine whether TTNtv and alcohol consumption in combination have any additional effect beyond the effects of TTNtv and alcohol separately. A p value ≤0.05 was considered statistically significant.

Statistical analyses were conducted in the R environment, version 3.0 (R Foundation for Statistical Computing, Vienna, Austria). All data and code required to reproduce these analyses are available online (24).

## Results

### Genetic contribution to ACM

To investigate the potential genetic contribution of cardiomyopathy genes to ACM, we examined cases for the presence of rare protein-altering variants in 9 genes known to cause DCM that were selected according to their previously reported excess of rare variants in DCM compared with control subjects (21). The frequency of variants was then compared between ACM cases, technically and ethnically matched DCM cases, and healthy volunteers (n = 141, n = 366, and n = 445, respectively) (cohort characteristics are shown in Table 1). We identified 20 distinct variants in 19 ACM cases involving 4 different genes (Table 2, Online Tables 2 and 3A and 3B). The prevalence of variants in ACM was significantly higher than in healthy volunteers (13.5% of ACM cases carry at least 1 variant vs. 2.9% of healthy volunteers; p = 0.000012), but not statistically different from the prevalence in the DCM cohort (19.4%; p = 0.12). The rate in healthy volunteers was as expected for the general population (Online Table 2). TTNtv accounted for the majority of variants detected in ACM cases (9.9%) and were found with a frequency similar to that seen in DCM (12.0%; p = 0.64), and significantly higher than in control subjects (0.7%; p = 4.4 × 10^−7^). In line with studies in DCM 10, 11, 12, 15, TTNtv found in ACM were in exons constitutively expressed in the heart and distributed across the gene (Online Figure 1) with 13 of 14 being novel (i.e., absent from previous DCM cases, healthy volunteers, and ExAC).

**Table 2:** Burden Analysis of Rare, Protein-Altering Variants in DCM-Related Genes Between Cohorts

	ACM (n = 141)	DCM (n = 366)	Healthy Volunteer (n = 445)	∗ACM vs. DCM	∗ACM vs. Healthy Volunteer	∗DCM vs. Healthy Volunteer
All genes	19 (13.5) (7.8%–19.1%)	71 (19.4) (15.3%–23.4%)	13 (2.9) (1.4%–4.5%)	0.12	1.2 × 10^−5^	5.4 × 10^−15^
TTNtv	14 (9.9) (5.0%–14.9%)	44 (12.0) (8.7%–15.4%)	3 (0.7) (0.0%–1.4%)	0.64	4.4 × 10^−7^	6.4 × 10^−12^
Genes other than *TTN*	6 (4.3) (0.9%–7.6%)	28 (7.7) (4.9%–10.4%)	10 (2.2) (0.9%–3.6%)	0.23	0.23	0.00035

Values are n (%) (95% confidence interval). The number of individual cases with a rare protein-altering variant is shown. *TTN* variants are TTNtv only; other variants are as described in Online Table 1. In ACM, 1 case had both a TTNtv and *LMNA* variant. In DCM, 1 case had both a TTNtv and a *BAG3* variant.

TTNtv = titin truncating variant; other abbreviations as in Table 1.

∗ Unadjusted p value (Fisher exact test).

We identified 6 ACM cases with rare, protein-altering variants in other DCM genes: 1 carrying a *BAG3* truncating variant previously reported in DCM (25) and classified as pathogenic for DCM under current variant interpretation guidelines from the American College of Medical Genetics and Genomics and the Association for Molecular Pathology (Online Tables 3A and 3B) (26), 1 carrying a novel BAG3 missense variant, 1 carrying both a TTNtv and a novel *LMNA* missense mutation, and 3 cases each carrying different *MYH7* variants.

**Figure 1:**
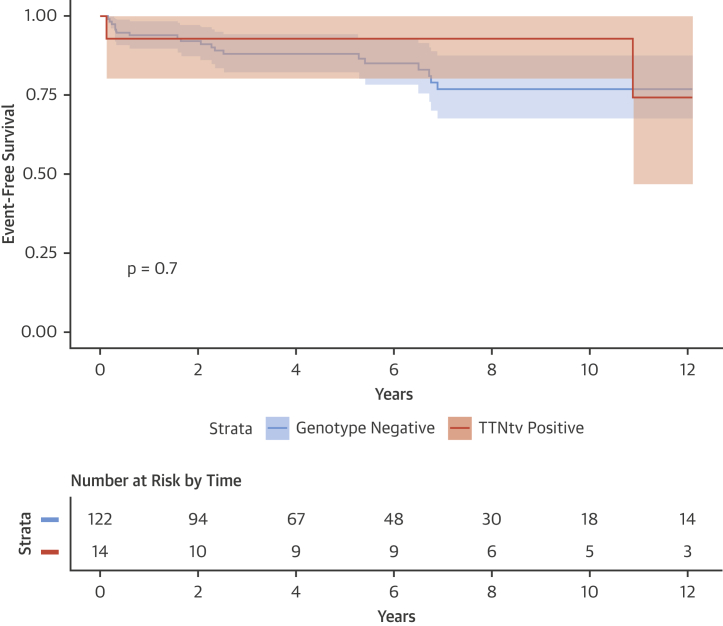
Survival Analysis of ACM Cases According to Genotype Survival curves show freedom from composite primary endpoint (all-cause mortality or cardiac transplant) between ACM cases stratified by genetic status: TTNtv positive (cases with a truncating variant in titin) or TTNtv negative. Event-free survival is measured from time of diagnosis. There is no significant difference between groups. Curves are compared using the log-rank test. ACM = alcoholic cardiomyopathy; TTNtv = titin truncating variant.

There were no detectable differences in phenotype or demographics between ACM cases with and without TTNtv (Table 3, Online Table 4), with the notable exception of family history of cardiomyopathy. On follow-up (overall mean follow-up = 5.9 ± 5.2 years), TTNtv status did not predict outcomes after reduction in alcohol intake and treatment for heart failure, with approximately one-half of all ACM cases showing LVEF recovery irrespective of TTNtv status (Table 3), and no detectable difference in event-free survival between the 2 groups (Figure 1).

**Table 3:** Characteristics of ACM Cases With and Without Titin Truncating Variants

	TTNtv (n = 14)	Genotype Negative (n = 122)	Other Variants (n = 5)	∗p Value
Alcohol, g/day	139.0 ± 68.7	136.0 ± 50.1	122.0 ± 34.6	0.85
Age at initial clinical assessment, yrs	49.4 ± 12.9	53.4 ± 9.6	58.8 ± 11.1	0.31
Initial left ventricular ejection fraction, %	25.1 ± 10.7	26.5 ± 9.1	30.4 ± 10.5	0.35
Initial left ventricular end-diastolic diameter, mm	63.2 ± 6.6	65.8 ± 9.2	68.4 ± 11.7	0.37
Male	13 (92.9)	120 (98.4)	5 (100.0)	0.28
Atrial fibrillation	5 (35.7)	41 (33.6)	3 (60.0)	1.00
Family history of cardiomyopathy	6 (42.9)	9 (7.4)	1 (20.0)	0.0012
Family history of sudden cardiac death	1 (7.1)	12 (9.8)	0 (0.0)	1.00
Outcomes	14	120	5	
Mean follow up period, yrs	8.3 ± 7.2	5.8 ± 4.9	5.5 ± 4.9	0.26
Death or transplant	3 (21.4)	19 (15.8)	3 (60.0)	0.96
Stable with recovery of left ventricular ejection fraction	7 (50.0)	55 (45.8)	0 (0.0)	0.78
Stable without recovery of left ventricular ejection fraction	4 (28.6)	46 (38.3)	2 (40.0)	0.57

Values are mean ± SD, n (%), or n. Age, left ventricular ejection fraction, left ventricular end-diastolic diameter, and atrial fibrillation taken at time of initial clinical assessment.

TTNtv = titin truncating variant.

∗ Unadjusted p values of TTNtv vs. genotype negative: Mann-Whitney *U* test for continuous variables, Fisher exact test for categorical variables, and Cox proportional hazard test for survival (death or transplant).

### Alcohol as a phenotypic modifier in DCM

**Figure 2:**
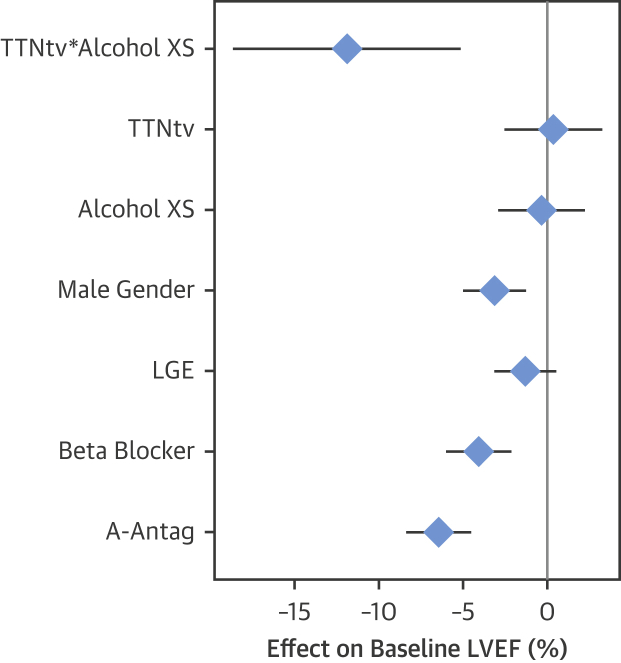
Alcohol and TTNtv Act in Combination, and Together Are Associated With a Lower Baseline LVEF in Patients With DCM Forest plot showing regression coefficient and 95% confidence intervals from the multivariable linear regression model evaluating the effects of TTNtv and excess alcohol consumption on baseline LVEF. The effect on LVEF is shown as absolute difference in LVEF (% = expressed as percentage of end-diastolic volume) between groups. A-Antag = aldosterone antagonist; Alcohol XS = excess alcohol consumption (binary variable indicating consumption >21 U/week for men, >14 U/week for women); LGE = late gadolinium enhancement (indicative of mid-wall fibrosis) on cardiovascular magnetic resonance; LVEF = left ventricular ejection fraction; TTNtv = presence of truncating variant in titin; TTNtv*Alcohol XS = interaction term representing individuals with both a TTNtv and a history of excess alcohol consumption.

Having established a genetic contribution to ACM in a proportion of cases, primarily driven by TTNtv, we investigated the interaction between TTNtv and alcohol consumption in the context of DCM but in the absence of prolonged and heavy alcohol consumption meeting criteria for ACM. A total of 111 of 716 DCM cases (15.5%) had a history of excess consumption (above U.K. guidelines, see the Methods section). These individuals were more likely to be male, and in univariate analyses had modestly reduced LVEF (median: 37.0% vs. 41.0%; p = 0.02) and right ventricular ejection fraction (median: 48.0% vs. 54.0%; p < 0.001) compared with DCM patients without a history of excess alcohol consumption (Online Table 5). A total of 83 DCM cases carried a TTNtv (11.6%). The presence of a TTNtv alone did not predict LVEF. In multivariable analysis accounting for covariate predictors of baseline LVEF, neither TTNtv nor excess alcohol consumption were significant predictors in isolation, but patients with both TTNtv and excess alcohol consumption (n = 13) had a statistically significant and biologically important reduction in LVEF **(**Figure 2, Online Tables 6A and 6B), with an 8.7% absolute reduction (95% confidence interval: −15.1 to −2.3; p = 0.007) compared with DCM with neither TTNtv nor excess alcohol consumption.

## Discussion

This study demonstrates an important genetic predisposition to ACM. We present a large series of ACM patients genotyped for variants in 9 genes associated with inherited DCM, and identified rare, protein-altering variants in 19 of 141 ACM cases (13.5%), a frequency significantly higher than that observed in healthy volunteers (2.9%; p = 0.000012) and population controls (ExAC, 4.3%; p = 0.0000059), but similar to that seen in DCM cases (19.4%; p = 0.12) (Central Illustration). Our findings demonstrate that in a proportion of ACM subjects, the disease has a genetic etiology.

**Central Illustration:**
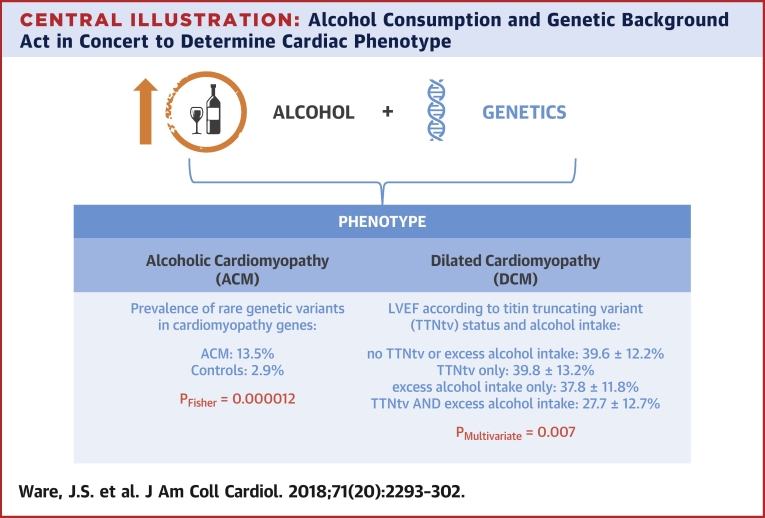
Alcohol Consumption and Genetic Background Act in Concert to Determine Cardiac Phenotype ACM patients exhibit a higher prevalence of rare variants in DCM-associated genes than control subjects. In DCM patients, neither the presence of a TTNtv nor excess alcohol consumption had a significant effect on baseline LVEF in isolation, but the combination was associated with a significantly lower baseline LVEF. Values shown are absolute ejection fraction in each group. The p value is derived from multivariate analysis. ACM = alcoholic cardiomyopathy; DCM = dilated cardiomyopathy; LVEF = left ventricular ejection fraction; TTNtv = titin truncating variant.

The data presented here indicate that patients with alcohol-related cardiomyopathy should undergo a 3-generation pedigree and should be considered for familial evaluation, such as clinical screening and genetic testing, to identify family members at risk for developing DCM (in line with current practice for idiopathic DCM).

**Figure 3:**
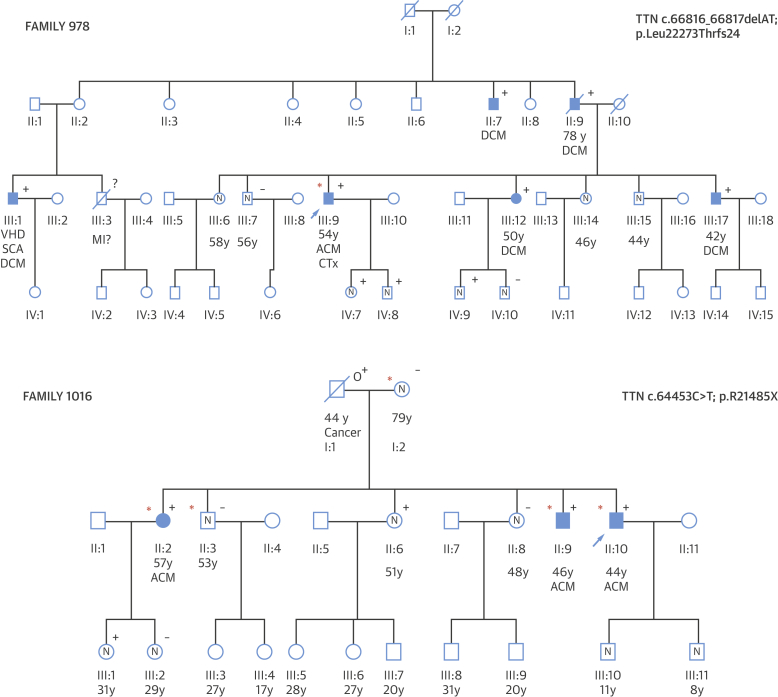
Family Pedigrees Illustrating Coexistence of ACM and DCM and the Combined Effect of Excessive Alcohol Consumption and Genetic Background **(Top)** Family 978: coexistence of ACM and DCM. The proband **(arrow)** was diagnosed with ACM and underwent cardiac transplantation. When genetic and clinical familial evaluation was performed, multiple individuals without excessive alcohol consumption were diagnosed with DCM and found to carry TTN truncating variants. **(Bottom)** Family 1016: combined effect of excessive alcohol consumption and genetic background. The proband **(arrow)** was diagnosed with ACM at age 44 years and was identified as carrying a TTNtv variant (TTN c.64453C>T; p.R21485X). One brother and 1 sister with prolonged heavy alcohol consumption **(red asterisk)** and TTNtv also show ACM. Two family members with TTNtv but no regular alcohol intake, and 2 individuals with prolonged heavy alcohol consumption but without TTNtv, did not show cardiac involvement. Standard pedigree notation is used: **squares and circles** indicate male and female subjects, respectively, a **strike-through** indicates a deceased individual, an **arrow** indicates the proband in each family, and **filled symbols** indicate affected individuals with ACM or DCM. **Symbols containing an N** represent individuals confirmed as unaffected. **+/−** symbols indicate genetic evaluation: **+** indicates carry TTNtv; **−** are noncarriers, **o+** are obligate carriers. **Red asterisks** indicate cases with documented prolonged heavy alcohol consumption. CTx = cardiac transplant; DCM = dilated cardiomyopathy; MI = myocardial infarction; SCA = sudden cardiac arrest; VHD = valvular heart disease; other abbreviations as in Figure 1.

An illustration of the utility of genetic management in ACM is shown in Figure 3, where familial evaluation identified several individuals with DCM, and molecular genetic testing enabled informed genetic counseling including reproductive advice. This reveals the importance of recognizing genetic disease and familial assessment, although future work will be needed to more fully understand the risk associated with genetic variants found in the absence of overt familial disease, to balance the costs and benefits associated with genetic testing and clinical surveillance, and to allow for fully informed genetic counseling.

We further identified a direct interaction between TTNtv and alcohol consumption in the context of typical DCM: cases with a TTNtv and excess alcohol consumption have a markedly reduced LVEF compared with those with low alcohol intake. Taken together, these 2 lines of evidence both support a model whereby alcohol and cardiac genotype interact, contributing both to the development of ACM and to disease severity in the context of DCM (Central Illustration). Although we acknowledge that many factors may contribute to the development of ACM, we identified an illustrative family where alcohol abuse and TTNtv were present in multiple relatives and probably acted in conjunction to promote disease expressivity in certain family members. In this pedigree, all 3 affected individuals both carried the TTNtv and reported prolonged heavy alcohol consumption, whereas 2 individuals who reported prolonged heavy alcohol consumption without the TTNtv and 3 individuals with the TTNtv but without excess alcohol consumption were all free from DCM (Figure 3).

There is still much to understand. The molecular mechanisms underlying ACM are not fully understood, and this study only explores some of the genetic factors that may influence susceptibility to cardiomyopathy on exposure to alcohol. Although there is strong evidence for an interaction between alcohol and TTNtv, there is much more to learn about the mechanisms underlying the variable penetrance of TTNtv. In some families with DCM, TTNtv appear highly penetrant and sufficient to cause disease in isolation, but TTNtv are also seen in approximately 1% of the general population (12), a level well above the prevalence of DCM and suggesting that other genetic or environmental factors contribute to the cardiomyopathic process (27).

The overall effect of alcohol on the occurrence of DCM is also difficult to assess, but previous reports have suggested that it may be involved in as many as 47% of cases (6), and a recent population-based study of >1.9 million U.K. individuals showed that 8.4% recorded drinking above the recommended safe levels (28). If this accurately reflects the proportion of the population with above-recommended alcohol intake, then we see a significantly higher exposure in our nonalcoholic DCM population (111 of 716 = 15.5%, p_binomial_ = 5 × 10^−10^). Together, these data suggest that alcohol alone, as well as in combination with genetic factors, may account for a substantial proportion of disease risk.

Additional environmental factors that may act in concert with TTNtv include viral myocarditis (29), nutritional deficiencies (30), recreational drug use (31), and certain drugs (32). Our data therefore also have wider potential implications both for lifestyle choices and for exploring the potential interaction of genetics with other environmental factors.

ACM has a poor prognosis, although somewhat better than DCM overall 2, 3. In the ACM cohort studied here, of the 139 cases with outcome data, 62 (44.6%) showed functional recovery following heart failure therapy and reduction in alcohol, 52 (37.4%) remained stable but without functional recovery, and 25 (17.9%) died or received a cardiac transplant, in agreement with recent studies 3, 16. We saw no difference between TTNtv and non-TTNtv cases with respect to outcome, with equivalent proportions showing improved cardiac function (50.0% vs. 45.8%, respectively) (Table 3), indicating that the presence of a TTNtv does not of itself preclude recovery. Functional recovery in DCM resulting from TTNtv has been previously reported both in severe end-stage failure requiring LVAD support (33) and in milder cases following medical therapy (34). Likewise, we observed no difference in survival analysis (freedom from death or cardiac transplantation) between TTNtv+ and TTNtv− groups.

### Study limitations

First, in the absence of a cohort with prolonged and heavy alcohol consumption but no cardiomyopathy, our comparison of ACM and healthy volunteers cannot formally exclude the possibility that TTNtv are associated with increased alcohol consumption, rather than the development of ACM on exposure to alcohol. However, this would seem highly unlikely and cannot explain the observed interaction between excess alcohol consumption and TTNtv as predictors of severity in an independent DCM cohort. Second, one might postulate that the individuals with coincident ACM and TTNtv simply represent conventional familial DCM: because prolonged heavy alcohol consumption is not uncommon in the population, a proportion of DCM cases will be exposed; thus, the TTNtv could be the causative driver, and the alcohol consumption a coincidental bystander. However, the positive cardiac response on reduction or cessation of alcohol points to an etiological role of alcohol in the disease process, and the observed synergistic interaction between genetic predisposition and environmental toxin in the DCM cohort once again points to a biological interaction.

Third, although the association between aggregated rare variation in this gene set and ACM can be robustly interpreted as demonstrating an etiological role, the interpretation of specific variants in individual patients often remains uncertain. Improvement in clinical variant interpretation would substantially improve the utility of genetic testing in cardiomyopathies more widely. We also restricted our analysis to robustly validated DCM genes with a published excess of rare variants in DCM compared with control subjects. We acknowledge that rare variants in other genes that might have a role in DCM may make a further contribution to a genetic predisposition to ACM.

Finally, self-reported alcohol consumption lacks precision and is likely under-reported, which, together with modest cohort size, limits our power to detect modest effect sizes on phenotype and outcome, to evaluate the contribution of genes that are more rarely variant, and to fully dissect the interactions between genetic and environmental influences.

## Conclusions

We have shown that TTNtv represent an important genetic predisposition to ACM, and that the combination of TTNtv and excess alcohol consumption is associated with worse LVEF in DCM patients. These findings support a model whereby alcohol and other environmental factors interact with genotype to determine the cardiac phenotype. Furthermore, based on our findings, familial evaluation and genetic testing should be considered in patients presenting with ACM.

Perspectives **COMPETENCY IN MEDICAL KNOWLEDGE:** Variants in DCM-associated genes are more frequent in patients with ACM than in the general population, and patients with DCM and TTNtv who drink alcohol excessively are more prone to decline in LVEF than those who drink less or lack these genetic variants.

**TRANSLATIONAL OUTLOOK:** Further studies are needed to understand how family history and genetic testing can be used to identify patients at risk of developing ACM, and effectively employed in counseling and other psychosocial interventions to reduce the incidence of this form of DCM.
