# Supplementary material for: Genetic Etiology for Alcohol-Induced Cardiac Toxicity
Source: J Am Coll Cardiol. 2018 May 22;71(20):2293–302. doi: 10.1016/j.jacc.2018.03.462 (PMC5957753; doi:10.1016/j.jacc.2018.03.462)
Supplement: Online Data [file mmc1.docx]

**A genetic etiology for alcohol-induced cardiac toxicity**

**SUPPLEMENTARY MATERIALS**

-

James S. Ware, MRCP, PhD,^a,b,c,^* Almudena Amor-Salamanca, MD,^d,^* Upasana Tayal, MRCP, PhD,^a,b,^* Risha Govind, MSC,^a,b,e,^* Isabel Serrano, MD,^f^ Joel Salazar-Mendiguchía, MD,^g,h^ Jose Manuel García-Pinilla, MD, PhD,^i,j^ Domingo A. Pascual-Figal, MD, PhD,^i,k^ Julio Nuñez, MD, PhD,^i,l^ Gonzalo Guzzo-Merello, MD, PhD,^d^  Emiliano Gonzalez-Vioque, PhD,^m^ Alfredo Bardaji, MD, PhD,^f^ Nicolas Manito, MD, PhD,^g^ Miguel A. López-Garrido, MD,^i,j^ Laura Padron-Barthe, PhD,^d,i^ Elizabeth Edwards, PhD,^a,b^ Nicola Whiffin, PhD,^a,b,c^ Roddy Walsh, MSc, PhD,^a,b^ Rachel J. Buchan, MSc,^a,b^ William Midwinter, BSc,^a,b^ Alicja Wilk, BSc,^a,b^ Sanjay Prasad, MD,^a,b^ Antonis Pantazis, MD,^b^ John Baski, MRCP, PhD,^b^ Declan P. O’Regan, MRCP, PhD,^c^ Luis Alonso-Pulpon, MD, PhD,^d,i^ Stuart A. Cook, MRCP, PhD,^a,c,n,o^ Enrique Lara-Pezzi, PhD,^i,p^

Paul J. Barton, PhD,^a,b,^* Pablo Garcia-Pavia, MD, PhD^d,i,q,^*

**Supplementary Methods**

**NGS Sequencing**

DNA samples in this study were sequenced using either the Illumina TruSight Cardio Sequencing Kit (which includes 174 genes associated with inherited cardiac conditions)^1^ on the Illumina NextSeq platform, or using a custom Agilent SureSelect panel with similar content and sequenced on the Life Technologies SOLiD 5500xl platform as described.^2^

Targeted libraries prepared using the TruSight Cardio kit (Illumina) were sequenced with paired-end reads of 150 bp on the NextSeq v2 platform (Illumina). Demultiplexing of sequence data was performed using NextSeq Control software or Bcl2FastQ conversion 2.16^3,4^ and resulting FastQ files subjected to quality control with the FastQC^5^ v.0.10.14. Low quality reads (Q<20, window_size 5) were trimmed using PrinSeq^6^ v0.20.4, and sequences aligned to the HG19 reference genome using BWA^7^ v0.7.10. Picard^8^ v1.115 and GATK^9^ v3.2-2 were used to mark duplicate reads and perform local realignment around indels and base quality score recalibration. Bases covered by at least 10 reads with a mapping quality ≥10 and base quality ≥20 were denoted as “callable”, i.e. adequately covered for variant calling with recommended GATK parameters. Variant calling was performed jointly with GATK HaplotypeCaller.

For samples analyzed using SOLiD5500XL, targeted DNA libraries were prepared according to manufacturers’ protocols before performing paired end sequencing. The SOLiD reads were aligned in color space using LifeScope™ v2.5.1 “Targeted re-sequencing” pipeline.^10^ The SOLiD Accuracy Enhancement Tool (SAET) was used to improve color call accuracy prior to mapping. Variants were called by the diBayes and SmallIndel packages in LifeScope software as well as GATK UnifiedGenotyper. Bases covered by at least 10 reads with a mapping quality ≥10 and base quality ≥20 were denoted as “callable”.

For all samples, variants were annotated with the Variant Effect Predictor (VEP)^11^ and according to defined transcripts for each gene (Supplementary Table 1) with truncating variants defined as those resulting in nonsense, frameshift, or essential splice site mutations (VEP consequence: frameshift variant, stop gained, splice donor variant, splice acceptor variant) and non-truncating variants defined as those resulting in missense variants and inframe indels (VEP consequence: missense variant, inframe deletion, inframe insertion).Cases used in burden testing were matched by ethnicity: we selected those self-reporting as Caucasian and confirmed ethnicity by principal component analysis (PCA) analyzed using PLINK v1.9 and HapMap3 as reference dataset. Any samples that fell outside the Caucasian cluster were removed from further analysis.

##
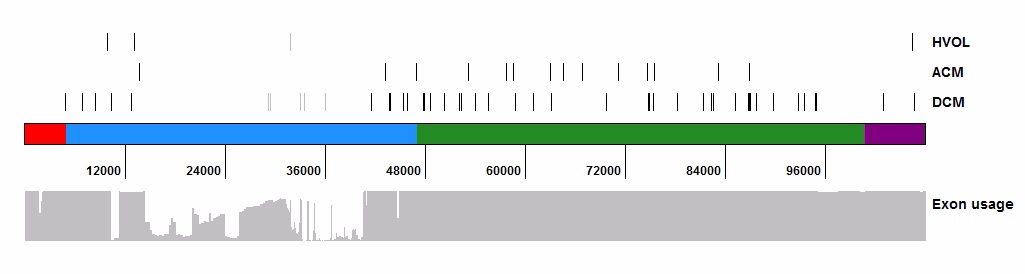


##

**Supplementary Figure 1 Distribution of TTNtv identified in ACM, DCM and healthy volunteers.**

A schematic of the *TTN* gene (LRG_391_t1/ENST00000589042) showing the corresponding protein domains (red, Z-disk; blue, I-band; green, A-band and purple, M-band) together with exon usage in human heart depicted as the proportion spliced-in (PSI) (range, 0 to 1; grey bars) as described.^2^ The position of TTNtv variants identified in constitutively expressed exons is shown for each of the cohorts of ACM (N=141), DCM (N=366) and healthy volunteers (HVOL, N=445) are shown by black vertical lines. Additional TTNtv identified in low expression exons are indicated as faded vertical bars for information.

## Supplementary Table 1 DCM genes and variant classes included in burden analysis.

| **Gene symbol** | **Non-Truncating** | **Truncating** | **Transcript ID** |
| --- | --- | --- | --- |
| BAG3 | YES | YES | ENST00000369085 |
| LMNA | YES | YES | ENST00000368300 |
| TCAP | YES | YES | ENST00000309889 |
| TNNC1 | YES | YES | ENST00000232975 |
| TNNT2 | YES | YES | ENST00000367318 |
| DSP | NO | YES | ENST00000379802 |
| SCN5A | NO | YES | ENST00000333535 |
| TTN | NO | YES | ENST00000589042 |
| MYH7 | YES | NO | ENST00000355349 |

## The genes assessed in this study are those with a demonstrated excess of rare variation in DCM clinical cohorts over ExAC reference samples, for either truncating or non-truncating variants,^12^ as well as BAG3 which has since been established as an important DCM gene.^13^ Genes thought to be primarily associated with pediatric DCM (TPM1 and VCL) were not included.^14^

**Supplementary Table 2 Summary of rare, protein-altering variants detected in ACM cases and matched DCM cases and healthy volunteers.**

|  | ACM (N=141) | DCM (N=366) | HVOL (N=445) | *ExAC (n=60706) |
| --- | --- | --- | --- | --- |
| **BAG3 non-tv** | 1 (0.7%) | 1 (0.3%) | 2 (0.4%) | 500 (0.84%) |
| **BAG3tv** | 1 (0.7%) | 0 (0%) | 0 (0%) | 4 (0.01%) |
| **DSPtv** | 0 (0%) | 3 (0.8%) | 0 (0%) | 42 (0.07%) |
| **LMNA non-tv** | 1 (0.7%) | 3 (0.8%) | 1 (0.2%) | 297 (0.58%) |
| **LMNAtv** | 0 (0%) | 2 (0.5%) | 0 (0%) | 8 (0.02%) |
| **MYH7 non-tv** | 3 (2.1%) | 12 (3.3%) | 6 (1.3%) | 815 (1.35%) |
| **SCN5A-tv** | 0 (0%) | 0 (0%) | 1 (0.2%) | 34 (0.06%) |
| **TCAP-tv** | 0 (0%) | 0 (0%) | 0 (0%) | 14 (0.03%) |
| **TCAP non-tv** | 0 (0%) | 1 (0.3%) | 0 (0%) | 120 (0.22%) |
| **TNNC1-tv** | 0 (0%) | 0 (0%) | 0 (0%) | 2 (0.003%) |
| **TNNC1 non-tv** | 0 (0%) | 0 (0%) | 0 (0%) | 33 (0.06%) |
| **TNNT2 tv** | 0 (0%) | 0 (0%) | 0 (0%) | 17 (0.03%) |
| **TNNT2 non-tv** | 0 (0%) | 6 (1.6%) | 0 (0%) | 120 (0.21%) |
| **TTNtv** | 14 (9.9%) | 44 (12%) | 3 (0.7%) | 484 (0.83%) |
| **All genes except TTN** | 6 (4.3%) | 28 (7.7%) | 10 (2.2%) | 2006 (3.48%) |
| **All genes** | 19 (13.5%) | 71 (19.4%) | 13 (2.9%) | 2490 (4.31%) |

The table gives the number (and %) of cases identified carrying rare, protein-altering variants identified in each gene, listed according to variant type: where truncating (tv) signifies stop-gain, frame shift or loss of essential splice site, and non-tv corresponds to missense variants and inframe indels. *For ExAC, the frequency was calculated by dividing the sum of rare protein-altering variants, by the mean of the total allele number at all protein-altering variant sites (in order to account for the variable coverage of whole exome sequencing in the ExAC dataset).

**Supplementary Table 3A**

List of individual variants detected in ACM cohort

| **Gene** | **HGVSg** | **HGVSc** | **HGVSp** | **Consequence** | **ExAC AF** | **Filtering AF** | **Filtering AF pop** | **ACMG class*** | **ACMG rules** |
| --- | --- | --- | --- | --- | --- | --- | --- | --- | --- |
| BAG3 | chr10: g.[121436753A>G] | c.1687A>G | p.Ser563Gly | missense variant | 0 | 0 | - | VUS | PM2,BP4 |
| BAG3 | chr10: g.[121435991C>T] | c.925C>T | p.Arg309Ter | stop gained | 0 | 0 | - | P | PVS1,PM2, PP1_strong |
| LMNA | chr1: g.[156106934A>G] | c.1519A>G | p.Ser507Gly | missense variant | 0 | 0 | - | VUS | PM2,PP2 |
| MYH7 | chr14: g.[23902931G>A] | c.11C>T | p.Ser4Leu | missense variant | 1.65E-05 | 5.00E-06 | nfe | VUS | PM2,PP2 |
| MYH7 | chr14: g.[23888715C>T] | c.3830G>A | p.Arg1277Gln | missense variant | 4.12E-05 | 2.10E-05 | sas | VUS | PM2,PP2 |
| MYH7 | chr14: g.[23884269G>A] | c.5494C>T | p.Arg1832Cys | missense variant | 4.94E-05 | 2.90E-05 | nfe | VUS | PM2,PP3,  PP2 |
| TTN | chr2: g.[179604345T>TA] | c.13614dupT | p.Asn4539Ter | frameshift variant | 0 | 0 | - | LP | PVS1_strong,PM2 |
| TTN | chr2: g.[179497710CCCAG>C] | c.43144_43147delCTGG | p.Leu14382ValfsTer2 | frameshift variant | 0 | 0 | - | LP | PVS1_strong,PM2 |
| TTN | chr2: g.[179483389C>A] | c.46888G>T | p.Gly15630Ter | stop gained | 0 | 0 | - | LP | PVS1_strong,PM2 |
| TTN | chr2: g.[179472344T>TAAGA] | c.53067_53070dupTCTT | p.Arg17691SerfsTer2 | frameshift variant | 0 | 0 | - | LP | PVS1_strong,PM2 |
| TTN | chr2: g.[179460363G>A] | c.57718C>T | p.Arg19240Ter | stop gained | 0 | 0 | - | LP | PVS1_strong,PM2 |
| TTN | chr2: g.[179458482A>T] | c.58545T>A | p.Tyr19515Ter | stop gained | 0 | 0 | - | LP | PVS1_strong,PM2 |
| TTN | chr2: g.[179453529CT>C] | c.62922delA | p.Val20975Ter | frameshift variant | 0 | 0 | - | LP | PVS1_strong,PM2 |
| TTN | chr2: g.[179450018G>A] | c.64453C>T | p.Arg21485Ter | stop gained | 2.48E-05 | 3.00E-05 | amr | LP | PVS1_strong,PM2 |
| TTN | chr2: g.[179445288AAT>A] | c.66816_66817delAT | p.Leu22273ThrfsTer24 | frameshift variant | 0 | 0 | - | LP | PVS1_strong,PM2 |
| TTN | chr2: g.[179439702GATGACATCACC>G] | c.71146_71156delGGTGATGTCAT | p.Gly23716HisfsTer6 | frameshift variant | 0 | 0 | - | LP | PVS1_strong,PM2 |
| TTN | chr2: g.[179436227C>A] | c.74632G>T | p.Glu24878Ter | stop gained | 0 | 0 | - | LP | PVS1_strong,PM2 |
| TTN | chr2: g.[179435424C>T] | c.75435G>A | p.Trp25145Ter | stop gained | 0 | 0 | - | LP | PVS1_strong,PM2 |
| TTN | chr2: g.[179427746GT>G] | c.83112delA | p.Glu27704AspfsTer19 | frameshift variant | 0 | 0 | - | LP | PVS1_strong,PM2 |
| TTN | chr2: g.[179424037C>G] | c.86821+1G>C | - | splice donor variant | 0 | 0 | - | LP | PVS1_strong,PM2 |

**ACMG class was determined using CardioClassifier^15^ followed by manual curation of segregation, de novo and functional data from the literature and ClinVar. P = pathogenic, LP = likely pathogenic, VUS = variant of uncertain significance*

*Filtering AF pop: nfe = non-Finnish European, sas = South Asian, amr = Latino*

**Supplementary Table 3B**

List of individual variants detected in DCM and HVOL cohorts

| **Cohort** | **Gene** | **HGVSg** | **HGVSc** | **HGVSp** | **Consequence** | **ExAC AF** | **Filtering AF** | **Filtering AF pop** |
| --- | --- | --- | --- | --- | --- | --- | --- | --- |
| DCM | BAG3 | chr10: g.[121436280C>T] | c.1214C>T | p.Ala405Val | missense variant | 8.24E-05 | 5.90E-05 | nfe |
| DCM | DSP | chr6: g.[7569571CAAGT>C] | c.1574+3_1574+6delAAGT | - | splice donor variant & coding sequence variant | 0 | 0 | - |
| DCM | DSP | chr6: g.[7581218GA>G] | c.4797delA | p.Gly1600AlafsTer2 | frameshift variant | 0 | 0 | - |
| DCM | DSP | chr6: g.[7585569CAGAA>C] | c.8077_8080delAAAG | p.Lys2693ProfsTer3 | frameshift variant | 0 | 0 | - |
| DCM | LMNA | chr1: g.[156085067T>G] | c.356+2T>G | - | splice donor variant | 0 | 0 | - |
| DCM | LMNA | chr1: g.[156104248C>T] | c.568C>T | p.Arg190Trp | missense variant | 0 | 0 | - |
| DCM | LMNA | chr1: g.[156104249G>A] | c.569G>A | p.Arg190Gln | missense variant | 0 | 0 | - |
| DCM | LMNA | chr1: g.[156104287G>A] | c.607G>A | p.Glu203Lys | missense variant | 0 | 0 | - |
| DCM | LMNA | chr1: g.[156104757T>G] | c.801T>G | p.Tyr267Ter | stop gained | 0 | 0 | - |
| DCM | MYH7 | chr14: g.[23899092C>T] | c.1030G>A | p.Glu344Lys | missense variant | 0 | 0 | - |
| DCM | MYH7 | chr14: g.[23898190C>T] | c.1381G>A | p.Asp461Asn | missense variant | 0 | 0 | - |
| DCM | MYH7 | chr14: g.[23894494C>T] | c.2420G>A | p.Arg807His | missense variant | 8.24E-06 | 0 | none |
| DCM | MYH7 | chr14: g.[23894036T>A] | c.2621A>T | p.Glu874Val | missense variant | 0 | 0 | - |
| DCM | MYH7 | chr14: g.[23893328G>A] | c.2710C>T | p.Arg904Cys | missense variant | 8.24E-06 | 0 | none |
| DCM | MYH7 | chr14: g.[23893327C>T] | c.2711G>A | p.Arg904His | missense variant | 0 | 0 | - |
| DCM | MYH7 | chr14: g.[23891455T>A] | c.3179A>T | p.Lys1060Met | missense variant | 0 | 0 | - |
| DCM | MYH7 | chr14: g.[23887584G>A] | c.4004C>T | p.Ser1335Leu | missense variant | 3.30E-05 | 5.00E-06 | nfe |
| DCM | MYH7 | chr14: g.[23887491G>A] | c.4097C>T | p.Ser1366Leu | missense variant | 2.47E-05 | 2.10E-05 | sas |
| DCM | MYH7 | chr14: g.[23887462C>T] | c.4126G>A | p.Glu1376Lys | missense variant | 0 | 0 | - |
| DCM | MYH7 | chr14: g.[23901919C>A] | c.431G>T | p.Gly144Val | missense variant | 0 | 0 | - |
| DCM | MYH7 | chr14: g.[23884966G>A] | c.5029C>T | p.Arg1677Cys | missense variant | 1.65E-05 | 0 | none |
| DCM | TCAP | chr17: g.[37821664C>T] | c.52C>T | p.Arg18Trp | missense variant | 0 | 0 | - |
| DCM | TNNT2 | chr1: g.[201333470G>A] | c.415C>T | p.Arg139Cys | missense variant | 0 | 0 | - |
| DCM | TNNT2 | chr1: g.[201333434G>A] | c.451C>T | p.Arg151Cys | missense variant | 0 | 0 | - |
| DCM | TNNT2 | chr1: g.[201331144G>A] | c.586C>T | p.Arg196Trp | missense variant | 0 | 0 | - |
| DCM | TNNT2 | chr1: g.[201331117G>A] | c.613C>T | p.Arg205Trp | missense variant | 0 | 0 | - |
| DCM | TNNT2 | chr1: g.[201331098ATCT>A] | c.629_631delAGA | p.Lys210del | inframe deletion | 0 | 0 | - |
| DCM | TNNT2 | chr1: g.[201331098ATCT>A] | c.629_631delAGA | p.Lys210del | inframe deletion | 0 | 0 | - |
| DCM | TTN | chr2: g.[179398383AT>A] | c.102958delA | p.Ile34320SerfsTer29 | frameshift variant | 0 | 0 | - |
| DCM | TTN | chr2: g.[179623709A>G] | c.10303+2T>C | - | splice donor variant | 8.24E-06 | 0 | none |
| DCM | TTN | chr2: g.[179393848CT>C] | c.106629delA | p.Ala35544ProfsTer2 | frameshift variant | 0 | 0 | - |
| DCM | TTN | chr2: g.[179605315CTG>C] | c.12643_12644delCA | p.Gln4215ValfsTer16 | frameshift variant | 0 | 0 | - |
| DCM | TTN | chr2: g.[179500850GC>G] | c.41447delG | p.Gly13816AlafsTer18 | frameshift variant | 0 | 0 | - |
| DCM | TTN | chr2: g.[179500825G>A] | c.41473C>T | p.Arg13825Ter | stop gained | 0 | 0 | - |
| DCM | TTN | chr2: g.[179497005GTGGTGTGTAGGCGC>G] | c.43602_43615delGCGCCTACACACCA | p.Gln14534HisfsTer23 | frameshift variant | 0 | 0 | - |
| DCM | TTN | chr2: g.[179495982AC>A] | c.43792delG | p.Val14598Ter | frameshift variant | 0 | 0 | - |
| DCM | TTN | chr2: g.[179486244G>A] | c.45307C>T | p.Arg15103Ter | stop gained | 0 | 0 | - |
| DCM | TTN | chr2: g.[179485525A>C] | c.45812T>G | p.Leu15271Ter | stop gained | 0 | 0 | - |
| DCM | TTN | chr2: g.[179641961CTTTCA>C] | c.4724_4728delTGAAA | p.Met1575SerfsTer6 | frameshift variant | 0 | 0 | - |
| DCM | TTN | chr2: g.[179482115G>T] | c.47697C>A | p.Cys15899Ter | stop gained | 0 | 0 | - |
| DCM | TTN | chr2: g.[179481846C>T] | c.47875+1G>A | - | splice donor variant | 0 | 0 | - |
| DCM | TTN | chr2: g.[179480145C>T] | c.48527G>A | p.Trp16176Ter | stop gained | 0 | 0 | - |
| DCM | TTN | chr2: g.[179477082G>A] | c.50170C>T | p.Arg16724Ter | stop gained | 0 | 0 | - |
| DCM | TTN | chr2: g.[179474001G>GAA] | c.52035_52036insTT | p.Leu17346PhefsTer4 | frameshift variant | 0 | 0 | - |
| DCM | TTN | chr2: g.[179473510C>CTTTCT] | c.52223_52227dupAGAAA | p.Asp17410ArgfsTer25 | frameshift variant | 0 | 0 | - |
| DCM | TTN | chr2: g.[179470140C>A] | c.53881+1G>T | - | splice donor variant | 0 | 0 | - |
| DCM | TTN | chr2: g.[179466192ATCCTGTC>A] | c.55525_55531delGACAGGA | p.Asp18509SerfsTer29 | frameshift variant | 0 | 0 | - |
| DCM | TTN | chr2: g.[179458293A>G] | c.58732+2T>C | - | splice donor variant | 0 | 0 | - |
| DCM | TTN | chr2: g.[179455521G>A] | c.60931C>T | p.Arg20311Ter | stop gained | 0 | 0 | - |
| DCM | TTN | chr2: g.[179453427G>A] | c.63025C>T | p.Arg21009Ter | stop gained | 0 | 0 | - |
| DCM | TTN | chr2: g.[179639647C>A] | c.6790+1G>T | - | splice donor variant | 0 | 0 | - |
| DCM | TTN | chr2: g.[179441341G>T] | c.69630C>A | p.Tyr23210Ter | stop gained | 0 | 0 | - |
| DCM | TTN | chr2: g.[179436099G>GT] | c.74759_74760insA | p.Ser24921LeufsTer11 | frameshift variant | 0 | 0 | - |
| DCM | TTN | chr2: g.[179436091TCCCTGGA>T] | c.74761_74767delTCCAGGG | p.Ser24921ThrfsTer23 | frameshift variant | 0 | 0 | - |
| DCM | TTN | chr2: g.[179435609G>A] | c.75250C>T | p.Arg25084Ter | stop gained | 0 | 0 | - |
| DCM | TTN | chr2: g.[179432675C>A] | c.78184G>T | p.Glu26062Ter | stop gained | 0 | 0 | - |
| DCM | TTN | chr2: g.[179432351CA>C] | c.78507delT | p.Gly26170ValfsTer3 | frameshift variant | 0 | 0 | - |
| DCM | TTN | chr2: g.[179429589AAGCATCTG>A] | c.81262_81269delCAGATGCT | p.Gln27088CysfsTer5 | frameshift variant | 0 | 0 | - |
| DCM | TTN | chr2: g.[179429538G>C] | c.81321C>G | p.Tyr27107Ter | stop gained | 0 | 0 | - |
| DCM | TTN | chr2: g.[179428619G>A] | c.82240C>T | p.Arg27414Ter | stop gained | 1.66E-05 | 0 | none |
| DCM | TTN | chr2: g.[179428345AT>A] | c.82513delA | p.Ile27505PhefsTer20 | frameshift variant | 0 | 0 | - |
| DCM | TTN | chr2: g.[179635210GCA>G] | c.8307_8308delTG | p.Ala2770HisfsTer4 | frameshift variant | 0 | 0 | - |
| DCM | TTN | chr2: g.[179426738CA>C] | c.84120delT | p.Ser28040ArgfsTer5 | frameshift variant | 0 | 0 | - |
| DCM | TTN | chr2: g.[179425769G>A] | c.85090C>T | p.Arg28364Ter | stop gained | 0 | 0 | - |
| DCM | TTN | chr2: g.[179424217TG>T] | c.86641delC | p.His28881ThrfsTer2 | frameshift variant | 0 | 0 | - |
| DCM | TTN | chr2: g.[179424036A>T] | c.86821+2T>A | - | splice donor variant | 8.28E-06 | 0 | none |
| DCM | TTN | chr2: g.[179423219C>T] | c.86967G>A | p.Trp28989Ter | stop gained | 0 | 0 | - |
| DCM | TTN | chr2: g.[179422272TC>T] | c.87716delG | p.Gly29239AspfsTer32 | frameshift variant | 0 | 0 | - |
| DCM | TTN | chr2: g.[179418515AG>A] | c.89216delC | p.Ala29739ValfsTer3 | frameshift variant | 0 | 0 | - |
| DCM | TTN | chr2: g.[179417876T>TC] | c.89750dupG | p.Val29918SerfsTer3 | frameshift variant | 0 | 0 | - |
| DCM | TTN | chr2: g.[179413670G>A] | c.92683C>T | p.Arg30895Ter | stop gained | 0 | 0 | - |
| DCM | TTN | chr2: g.[179412874C>T] | c.93479G>A | p.Trp31160Ter | stop gained | 0 | 0 | - |
| DCM | TTN | chr2: g.[179411432TGA>T] | c.94721_94722delTC | p.Leu31574GlnfsTer2 | frameshift variant | 0 | 0 | - |
| DCM | TTN | chr2: g.[179411199AATCGGGC>A] | c.94852_94858delGCCCGAT | p.Ala31618TyrfsTer37 | frameshift variant | 0 | 0 | - |
| HVOL | BAG3 | chr10: g.[121429598G>A] | c.416G>A | p.Arg139Gln | missense variant | 1.65E-05 | 0 | none |
| HVOL | BAG3 | chr10: g.[121431768G>A] | c.509G>A | p.Arg170Gln | missense variant & splice region variant | 4.94E-05 | 8.30E-05 | sas |
| HVOL | LMNA | chr1: g.[156106126C>T] | c.1279C>T | p.Arg427Cys | missense variant | 4.12E-05 | 2.00E-05 | nfe |
| HVOL | MYH7 | chr14: g.[23894555G>A] | c.2359C>T | p.Arg787Cys | missense variant | 5.77E-05 | 3.90E-05 | nfe |
| HVOL | MYH7 | chr14: g.[23894555G>A] | c.2359C>T | p.Arg787Cys | missense variant | 5.77E-05 | 3.90E-05 | nfe |
| HVOL | MYH7 | chr14: g.[23890202C>T] | c.3301G>A | p.Gly1101Ser | missense variant | 6.59E-05 | 3.90E-05 | nfe |
| HVOL | MYH7 | chr14: g.[23889323C>T] | c.3457G>A | p.Ala1153Thr | missense variant | 8.30E-06 | 0 | none |
| HVOL | MYH7 | chr14: g.[23884670A>T] | c.5203T>A | p.Ser1735Thr | missense variant | 3.30E-05 | 2.00E-05 | nfe |
| HVOL | MYH7 | chr14: g.[23899870T>A] | c.898A>T | p.Met300Leu | missense variant & splice region variant | 8.24E-06 | 0 | none |
| HVOL | SCN5A | chr3: g.[38591991G>A] | c.5872C>T | p.Arg1958Ter | stop gained | 6.61E-05 | 7.30E-05 | amr |
| HVOL | TTN | chr2: g.[179394786C>A] | c.106432G>T | p.Glu35478Ter | stop gained | 0 | 0 | - |
| HVOL | TTN | chr2: g.[179604901TG>T] | c.13058delC | p.Pro4353GlnfsTer14 | frameshift variant | 0 | 0 | - |
| HVOL | TTN | chr2: g.[179629515G>A] | c.9727C>T | p.Gln3243Ter | stop gained | 0 | 0 | - |

*Filtering AF pop: nfe = non-Finnish European, sas = South Asian, amr = American*

## Supplementary Table 4 Clinical characteristics of ACM cohort based on genotype.

|  | TTNtv (N=14) | Genotype Neg (N=122) | Other Variants (N=5) | TTNtv (NA's) | Genotype Neg  (NA's) | Other Variants (NA's) | *P value |
| --- | --- | --- | --- | --- | --- | --- | --- |
| **Initial NYHA class 1/2/3/4** | 2/7/5/0 | 18/59/27/18 | 1/2/1/1 | 0 | 0 | 0 | 0.7 |
| **Initial left ventricular end-diastolic diameter - mm** | 63.2 ± 6.6 | 65.8 ± 9.2 | 68.4 ± 11.7 | 0 | 5 | 0 | 0.37 |
| **Final left ventricular end-diastolic diameter - mm** | 55.9 ± 9.3 | 58.6 ± 9.5 | 53.3 ± 2.5 | 3 | 18 | 2 | 0.44 |
| **Initial left ventricular ejection fraction - %** | 25.1 ± 10.7 | 26.5 ± 9.1 | 30.4 ± 10.5 | 0 | 0 | 0 | 0.35 |
| **Final left ventricular ejection fraction - %** | 41.9 ± 13.6 | 42.2 ± 14.9 | 40.2 ± 17.5 | 0 | 4 | 0 | 0.94 |
| **Recovery of left ventricular ejection fraction - no. (%)** | 7 (50%) | 55 (45.8%) | 0 (0%) | 0 | 2 | 0 | 0.78 |
| **Death - no. (%)** | 1 (7.1%) | 8 (6.6%) | 2 (40%) | 0 | 0 | 0 | 1 |
| **Transplant - no. (%)** | 2 (14.3%) | 11 (9%) | 1 (20%) | 0 | 0 | 0 | 0.62 |
| **Non-drinker (last assessment) – no. (%)** | 10 (71.4%) | 69 (56.6%) | 4 (80%) | 0 | 0 | 0 | 0.39 |
| **CRT - no. (%)** | 2 (14.3%) | 20 (16.4%) | 0 (0%) | 0 | 0 | 0 | 1 |
| **QRS greater than 120ms - no. (%)** | 3 (21.4%) | 38 (31.4%) | 3 (60%) | 0 | 1 | 0 | 0.55 |
| **ICD- no. (%)** | 7 (50%) | 30 (24.6%) | 0 (0%) | 0 | 0 | 0 | 0.058 |
| **Smoker- no. (%)** | 4 (28.6%) | 55 (45.1%) | 2 (40%) | 0 | 0 | 0 | 0.27 |
| **Hypertension- no. (%)** | 4 (28.6%) | 67 (54.9%) | 1 (20%) | 0 | 0 | 0 | 0.089 |
| **Dyslipidemia- no. (%)** | 6 (42.9%) | 50 (41%) | 1 (20%) | 0 | 0 | 0 | 1 |
| **Diabetes mellitus - no. (%)** | 5 (35.7%) | 36 (29.5%) | 2 (40%) | 0 | 0 | 0 | 0.76 |
| **COPD - no. (%)** | 3 (21.4%) | 33 (27%) | 2 (40%) | 0 | 0 | 0 | 0.76 |

*Unadjusted P values of TTNtv vs Genotype negative (Mann–Whitney *U* test for continuous variables and NYHA class, Fisher’s exact test was used for all other categorical variables). NA = number of cases with missing data per category.

## Supplementary Table 5

## Clinical characteristics of idiopathic DCM patients with and without a history of excess alcohol consumption.

| Variable | Moderate alcohol excess | No alcohol excess | P value |
| --- | --- | --- | --- |
|  | N=111 | N=605 |  |
| Age (years) | 53.9 ± 13.1 | 53.4 ± 14.5 | 0.77 |
| Gender Male | 104 (93.7%) | 365 (60.3%) | <0.0001 |
| Baseline LVEF (%) | 37.00 [27.50, 47.00] | 41.00 [30.00, 49.00] | 0.02 |
| Baseline RVEF (%) | 48.00 [39.00, 56.00] | 54.00 [44.50, 62.00] | <0.001 |
| LGE | 47 (42.3%) | 203 (33.6%) | 0.08 |
| TTNtv | 13 (11.7%) | 70 (11.6%) | 1.00 |

Continuous data are expressed as mean ± standard deviation or median [interquartile range]. Continuous variables were compared using Mann-Whitney or t-tests, as appropriate. Categorical data are expressed as number and percentages, and compared using Fisher’s exact test. LVEF = left ventricular ejection fraction; RVEF = right ventricular ejection fraction; LGE = late gadolinium enhancement mid wall fibrosis; TTNtv = titin truncating variant.

**Supplementary Table 6A**

**Regression analysis evaluating TTNtv and excess alcohol consumption as predictors of LVEF.**

|  | **Unadjusted analysis** | | | **Adjusted analysis*** | | |
| --- | --- | --- | --- | --- | --- | --- |
| **Variable** | **Estimate**  **(change in LVEF, %)** | **95% confidence intervals** | **P value** | **Estimate**  **(change in LVEF, %)** | **95% confidence intervals** | **P value** |
| Baseline:  No TTNtv or excess alcohol consumption | 0 | - | - | 0 | - | - |
| TTNtv, no excess alcohol consumption | 0.2 | -2.8 to 3.3 | 0.87 | 0.3 | -2.5 to 3.2 | 0.83 |
| Excess alcohol consumption , no TTNtv | -1.8 | -4.4 to 0.87 | 0.18 | -0.3 | -2.9 to 2.2 | 0.79 |
| TTNtv and excess alcohol consumption | -11.9 | -18.6 to  -5.1 | 0.0006 | -8.7 | -15.1 to -2.3 | 0.007 |
| TTNtv*excess alcohol consumption interaction§ | -10.3 | -18.1 to  -2.6 | 0.009 | -8.8 | -15.9 to -1.4 | 0.02 |
| *Male gender* | *-3.6* | *-5.5 to -1.7* | *0.0002* | *-3.1* | *-5.0 to 1.3* | *0.001* |
| *Beta blocker use* | *-6.4* | *-8.3 to -4.5* | *<0.00001* | *-4.1* | *-6.0 to -2.1* | *0.00004* |
| *Aldosterone Antagonist use* | *-7.9* | *-9.7 to -6.2* | *<0.00001* | *-6.5* | *-8.3 to -4.6* | *<0.00001* |
| *Mid wall fibrosis present* | *-3.2* | *-5.1 to -1.3* | *0.001* | *-1.3* | *-3.1 to 0.5* | *0.15* |

Table shows unadjusted univariable and adjusted multivariable analyses of the effect of TTNtv and excess alcohol consumption on baseline left ventricular ejection fraction.

*Adjusted for gender, prognostic medication (beta blocker use and aldosterone antagonist use), and the presence of mid wall fibrosis (late gadolinium enhancement on CMR); univariate and multivariate regression coefficients shown in italics.

§ i.e. the effect of TTNtv and excess alcohol consumption compared to either TTNtv alone or excess alcohol consumption alone.

**Supplementary Table 6B**

|  | **Mean LVEF (sd)** |
| --- | --- |
| Male | 37.9 (12.6) |
| Female | 41.5 (11.7) |
| LGE + | 37.1 (12.5) |
| LGE- | 40.2 (12.2) |
| Beta blocker + | 37.2 (12.2) |
| Beta blocker - | 43.6 (11.6) |
| Aldosterone Antagonist + | 34.0 (12.0) |
| Aldosterone Antagonist - | 42.0 (11.6) |
| TTNtv+ / excess alcohol consumption + | 27.7 (12.7) |
| TTNtv+ / excess alcohol consumption - | 39.8 (13.2) |
| TTNtv- / excess alcohol consumption + | 37.8 (11.8) |
| TTNtv- / excess alcohol consumption - | 39.6 (12.2) |

Table shows mean left ventricular ejection fraction (LVEF) and standard deviation (sd) for groups of patients stratified by variables included in the regression analysis.

**References to supplementary materials**

1. Pua CJ, Bhalshankar J, Miao K, et al. Development of a Comprehensive Sequencing Assay for Inherited Cardiac Condition Genes. *J Cardiovasc Transl Res* 2016; **9**(1): 3-11.

2. Roberts AM, Ware JS, Herman DS, et al. Integrated allelic, transcriptional, and phenomic dissection of the cardiac effects of titin truncations in health and disease. *Sci Transl Med* 2015; **7**(270): 270ra6.

3. <http://supportilluminacom/sequencing/sequencing_software/miseq_reporter/downloadsilmn>.

4. <http://supportilluminacom/sequencing/sequencing_instruments/miseq/downloadsilmn>.

5. <http://wwwbioinformaticsbabrahamacuk/projects/fastqc/>.

6. Schmieder R, Edwards R. Quality control and preprocessing of metagenomic datasets. *Bioinformatics* 2011; **27**(6): 863-4.

7. Li H, Durbin R. Fast and accurate long-read alignment with Burrows-Wheeler transform. *Bioinformatics* 2010; **26**(5): 589-95.

8. <http://picardsourceforgenet>

9. McKenna A, Hanna M, Banks E, et al. The Genome Analysis Toolkit: a MapReduce framework for analyzing next-generation DNA sequencing data. *Genome Res* 2010; **20**(9): 1297-303.

10. <http://wwwlifetechnologiescom/us/en/home/technical-resources/software-downloads/lifescope-genomic-analysis-softwarehtml>.

11. McLaren W, Gil L, Hunt SE, et al. The Ensembl Variant Effect Predictor. *Genome Biol* 2016; **17**(1): 122.

12. Walsh R, Thomson KL, Ware JS, et al. Reassessment of Mendelian gene pathogenicity using 7,855 cardiomyopathy cases and 60,706 reference samples. *Genet Med* 2017; **19**(2): 192-203.

13. Villard E, Perret C, Gary F, et al. A genome-wide association study identifies two loci associated with heart failure due to dilated cardiomyopathy. *Eur Heart J* 2011; **32**(9): 1065-76.

14. Pugh TJ, Kelly MA, Gowrisankar S, et al. The landscape of genetic variation in dilated cardiomyopathy as surveyed by clinical DNA sequencing. *Genet Med* 2014; **16**(8): 601-8.

15. Whiffin N, Walsh R, Govind R, et al. CardioClassifier: demonstrating the power of disease- and gene-specific computational decision support for clinical genome interpretation. *Genet Med* 2018; online publication doi:10.1038/gim.2017.258.
